# Antimicrobial spectrum against wound pathogens and cytotoxicity of star-arranged poly-l-lysine-based antimicrobial peptide polymers

**DOI:** 10.1099/jmm.0.001886

**Published:** 2024-09-13

**Authors:** Aaron Doherty, Robert Murphy, Andreas Heise, Fidelma Fitzpatrick, Deirdre Fitzgerald-Hughes

**Affiliations:** 1 Department of Clinical Microbiology, Royal College of Surgeons in Ireland University of Medicine and Health Sciences, Education and Research Centre, Beaumont Hospital, Dublin 9, Ireland; 2 Department of Clinical Microbiology, Beaumont Hospital, Dublin 9, Ireland; 3 Department of Clinical Microbiology, Cork University Hospital, Wilton, Cork, Ireland; 4 Department of Chemistry, Royal College of Surgeons in Ireland University of Medicine and Health Science, 123 St. Stephen’s Green, Dublin 2, Ireland

**Keywords:** anti-bacterial agents, antibiotic resistance, antimicrobial peptides, cationic host defense peptides, cytotoxicity, drug resistance, Wound infection

## Abstract

**Introduction.** As growing numbers of patients are at higher risk of infection, novel topical broad-spectrum antimicrobials are urgently required for wound infection management. Robust pre-clinical studies should support the development of such novel antimicrobials.

**Gap statement.** To date, evidence of robust investigation of the cytotoxicity and antimicrobial spectrum of activity of antimicrobial peptides (AMP)s is lacking in published literature. Using a more clinical lens, we address this gap in experimental approach, building on our experience with poly-l-lysine (PLL)-based AMP polymers.

**Aim.** To evaluate the *in vitro* bactericidal activity and cytotoxicity of a PLL-based 16-armed star AMP polymer, designated 16-PLL_10_, as a novel candidate antimicrobial.

**Methods.** Antimicrobial susceptibilities of clinical isolates and reference strains of ESKAPE (*Enterococcus* spp., *Staphylococcus aureus, Klebsiella pneumoniae, Acinetobacter baumannii, Pseudomonas aeruginosa*, *Enterobacter* spp.) pathogens, to 16-PLL_10_ were investigated. Human erythrocyte haemolysis and keratinocyte viability assays were used to assess toxicity. Modifications were made to 16-PLL_10_ and re-evaluated for improvement.

**Results.** Minimum bactericidal concentration of 16-PLL_10_ ranged from 1.25 µM to ≥25 µM. At 2.5 µM, 16-PLL_10_ was broadly bactericidal against ESKAPE strains/wound isolates. Log-reduction in colony forming units (c.f.u.) per millilitre after 1 h, ranged from 0.3 (*E. cloacae*) to 5.6 (*K. pneumoniae*). At bactericidal concentrations, 16-PLL_10_ was toxic to human keratinocyte and erythrocytes. Conjugates of 16-PLL_10_, Trifluoroacetylated (TFA)−16-PLL_10_, and Poly-ethylene glycol (PEG)ylated 16-PLL_10_, synthesised to address toxicity, only moderately reduced cytotoxicity and haemolysis.

**Conclusions.** Due to poor selectivity indices, further development of 16-PLL_10_ is unlikely warranted. However, considering the unmet need for novel topical antimicrobials, the ease of AMP polymer synthesises/modification is attractive. To support more rational development, prioritising clinically relevant pathogens and human cells, to establish selective toxicity profiles *in vitro*, is critical. Further characterisation and discovery utilising artificial intelligence and computational screening approaches can accelerate future AMP nanomaterial development.

**Impact Statement**

Poly-l-lysine (PLL)-based nanomaterials are antimicrobial peptides (AMP)s, with distinctive physical and chemical properties, making them attractive for biomedical applications, e.g. drug delivery. In the face of diminishing antibiotic discovery pipelines, they are noted for their antimicrobial properties, biocompatibility, biodegradability and ease/low-cost of synthesis and modification. However, their pre-clinical *in vitro* evaluation regularly assumes broad-spectrum activity based on data for one or two bacteria, and reiterates claims of human cell biocompatibility despite a paucity of cytotoxicity data pertaining to human cell lines. In this study, one such structure, 16-PLL_10_ (a star shaped structure with 16 arms of 10 lysine residues arranged around a central core), killed a broad range of clinically relevant bacteria. Its compelling bactericidal activity against pathogens that frequently infect wounds, including those with the highest rates of antimicrobial resistance, supported its potential for further development as a topical agent for wound infections. However, these nanomaterials were unfavourably toxic to human red blood cells and laboratory-grown human skin cells, at bactericidal concentrations. Only moderate improvement in biocompatibility indicators was apparent with further chemical modification. The grave healthcare and societal need for antibiotic alternatives demands robust investigation of promising AMP nanomaterials as future therapeutic agents which must include clinically-focused improvements in their selective toxicity. To fully exploit the potential of lead structures for clinical progression, early phase identification of their antimicrobial properties and activity spectrum are important, but where discovered, their potential human cell cytotoxicity should also be disclosed so that rationalised modifications can be advanced to address these. The generation and distribution of data pertaining to the preclinical characterisation of AMPs, (both positive and negative results), becomes increasingly relevant in providing training datasets for machine learning as the role of artificial intelligence (AI) in antimicrobial drug discovery comes to the fore.

## Data Summary

The authors confirm all supporting data and protocols have been provided within the article or through supplementary data files.

## Introduction

Wound infections are complex with unpredictable clinical courses associated with significant patient morbidity and increased healthcare costs. Antimicrobial resistance (AMR) makes infection management more challenging [1]. Patients with wound infections are frequently hospitalized, and commonly require multiple, prolonged antimicrobials courses for polymicrobial infections, further promoting AMR development [2]. Currently, topical antimicrobials are not recommended for treatment of wound infections due to poor efficacy, significant side-effect profile, propensity for rapid AMR development and importantly, a paucity of robust randomized controlled trials [3,5]. Nonetheless, AMPs and AMP polymers are emerging as potential topical alternatives for wound infection management to address this unmet clinical need. While cytotoxicity appears to be mitigated by developing AMPs into cationic, amphiphilic polymers, there is high variability in the approaches used to establishing biocompatibility [6].

AMPs are cationic amphipathic peptides, which unlike most conventional antibiotic classes, target cell membranes [7,9]. Biologically, AMPs are part of the innate immune system of numerous organisms. These natural structures have been chemically modified to produce various AMP mimetics, synthetic AMPs and analogues. Antimicrobial polypeptides are similar to AMPs but polymeric, larger in size, and more complex. They have built-in three-dimensional macromolecular structure which endows them with additional properties. For example, structurally nanoengineered antimicrobial peptide polymers (SNAPPs) gained attention as delivery vehicles for intracellular therapeutics including non-translational RNAs and DNAs, chemotherapeutics and antimicrobial agents [10,12]. ome of these structures were found to have intrinsic antimicrobial properties [13,16]. A publication in 2016, highlighting the bactericidal activity and low cytotoxicity of SNAPPs comprising heteropolymeric AMPs of lysine and valine residues arranged around a central poly(amidoamine) dendritic core, prompted wider interest in the antimicrobial applications of star-shaped AMPs [13]. The authors concluded that the star arrangement, gave superior broad-spectrum antimicrobial activity compared to equivalent linear AMPs.

Poly-l-lysine (PLL) based star-shaped AMPs have cell-penetrating, drug-delivery properties for bone regeneration [17]. These polymeric structures have PLL ‘arms’ branching from a central poly(propylene imine) core, with high loading capabilities with other molecules, e.g. gene cargos [15,18]. We recently showed PLL-based star AMPs had anti-staphylococcal and anti-pseudomonal activity and potent anti-biofilm properties [16].

Generally, *in vitro* investigation of novel antimicrobials aims to establish potent and selective toxicity (predictive of a broad therapeutic index), with antimicrobial spectra clinically relevant to the envisioned application. However, with some exceptions, the AMPs described in the literature regularly claim broad-spectrum activity, based on one or two reference organisms (e.g. Gram-positive and Gram-negative bacterial species) or lack of human cell cytotoxicity based on non-human cell lines [13,15]. In this study, we aimed to establish the potential clinical applicability of a PLL-based AMP polymer, 16-PLL_10_ for development as a topical antimicrobial for wound infections. Bactericidal spectrum investigation was extended to clinically-important wound pathogens, including ESKAPE (*Enterococcus* spp., *Staphylococcus aureus, Klebsiella pneumoniae, Acinetobacter baumannii, Pseudomonas aeruginosa*, *Enterobacter* spp.) reference strains and clinical isolates from wound infections. Cytotoxicity to human keratinocytes and erythrocytes was investigated and the effect of synthetic modifications; trifluoroacetylated 16-PLL_10_, and PEGylated 16-PLL_10_ on selective toxicity was assessed.

## Methods

### AMP synthesis

Polymeric AMPs with star arrangements of PLL were provided as lyophilised powders. Base materials and PLLs were synthesised via *N*-carboxyanhydride ring-opening polymerisation (NCA ROP) as previously described [15,16]. Molecular weights were determined by gel permeation chromatography and ^1^H Nuclear Magnetic Resonance (NMR) spectroscopy. Trifluoroacetic acid (TFA) counterion mass, lysine repeat unit mass and dendrimer core were considered contributory. The parent PLL (16-PLL_10_) had a poly(propylene imine) core with 16 polymeric l-lysine arms, 10 l-lysine subunits per arm, *Mn*=40 400 g mol^−1^. Two further modifications were made as follows; TFA-16-PLL_10_, ~25% of l-lysine repeat units were conjugated with TFA functional groups (7.25 l-lysine/2.75 TFA-l-lysine per arm, *Mn*=39 600 g mol^−1^) and 2.5% of l-lysine repeats were conjugated with polyethylene glycol (*Mn* 5000 g mol^−1^), PEGylated-16-PLL10 (*Mn*=60 400 g mol^−1^). Structural features are summarised in Table 1.

**Table 1.** 16-Poly-l-Lysine (16-PLL^_10_^) and modified versions investigated

### Bacterial strains and isolates

Reference strains were sourced from the National Collection of Type Cultures (NTCC) United Kingdom Health Security Agency (UKHSA) or the American Type Culture Collection (ATCC) and selected to represent ESKAPE pathogens. Where possible, AMR and fully antibiotic susceptible strains were included (Table S1, available in the online Supplementary Material). Clinical isolates (Table S1) were collected from November 2022–January 2023, Microbiology Laboratory, Beaumont Hospital, Dublin Ireland and were confirmed to species level by Matrix Associated Laser Ionisation Desorption Time-of-Flight Mass Spectrometry (MALDI-TOF MS).

### Antimicrobial susceptibility testing

Bacteria suspensions were prepared in sterile phosphate buffered saline (PBS) from colonies grown on Mueller-Hinton (MH) at 37 °C overnight. Suspensions were adjusted to 0.5 McFarland standard using a DensiCHEK Plus device (BioMérieux, Marcy l'Etoile, France) and diluted 1/50 in PBS (Approximately 1×10^6^ colony forming units [c.f.u.] ml^−1^). Assays contained 10 µl diluted bacterial suspensions, variable stock PLLs, and buffer (10 mM potassium phosphate, 0.2% Bovine Serum Albumin [BSA]) to a final volume of 100 µl. Buffer replaced AMPs for controls. Assays were incubated for 1 h at 37 °C, shaking at 150 r.p.m. (Tiger ECO 260, Labwit orbital incubator). Serial 1/10 dilutions were prepared in PBS. Using the method of Miles *et al*., 20 µl × three droplets from dilutions were plated on MH agar and incubated at 37 °C overnight [19]. Colonies were enumerated from treated and control assays. Log differences in c.f.u. ml^−1^ relative to control assays, were calculated.

For minimum bactericidal concentration (MBC) determination, bacterial suspensions were prepared in MH broth (0.5 McFarland) and diluted 1/1000 (approximately 1×10^4^ cells ml^−1^). For each PLL/bacterial isolate combination, 96 well plates were prepared, containing diluted inoculum and PLLs from 0 to 40 µg ml^−1^, prepared from stock concentrations in 0.2% BSA/0.01% acetic acid. Plates were incubated at 37 °C overnight and 3×20 µl samples were pipetted from each well onto quadrants of MH agar plates. Plates were air-dried, incubated at 37 °C overnight and MBC determined as the lowest concentration at which no colonies were observed.

### Metabolic assay using human keratinocytes in culture

Human keratinocyte (HaCaT) cells were cultured in Dulbecco’s modified Eagles medium (DMEM) supplemented with 10% foetal bovine serum (FBS). Wells were seeded with 3×10^4^ cells per well and following overnight adherence, cells washed with PBS and incubated with PLLs (0–10 µM), or 2% Triton X-100 (control) for 18 h. Following washing, cell viability was estimated by incubating with 100 µl 0.5 mg ml^−1^ 3-(4,5-dimethylthiazol-2-yl)−2-,5-diphenyltetrazolium bromide (MTT) in DMEM in the dark for 3–4 h at 37 °C before replacement of MTT with 100 µl dimethyl sulfoxide (DMSO). Plates were agitated in the dark on an orbital shaker for 5–10 min and absorbance at 595 nm was measured (VICTOR X3 2030 Multilabel Reader, PerkinElmer). Percentage cell-viability was estimated with respect to untreated controls and IC_50_ values were determined using GraphPad Prism, Version 9.3.1.

### Haemolysis of human erythrocytes

A modification of the haemolysis assays previously described was used [20,21]. Aliquots (5 ml) of healthy human volunteer blood were centrifuged at 500 ***g*** for 5 min. Supernatants were aspirated, and erythrocyte-rich pellets resuspended to original volume with PBS. Following two washes, pellets were resuspended to five times the original volume of blood collected. In a 96 well plate, 50 µl of the erythrocyte suspension was mixed with 50 µl of two-fold dilutions of PLLs (10μM to 0.0195 µM). Wells containing erythrocytes and 2% Triton X-100 (positive control, 100% haemolysis), PBS (negative controls, 0% haemolysis) were included. After 18 h incubation at 37 °C, plates were centrifuged, 500 ***g*** for 5 min. Supernatants were removed to fresh 96 well plates, and absorbance (405 nm) measured (VICTOR X3 2030 Multilabel Reader, PerkinElmer). Percentage haemolysis was calculated with reference to controls.

## Results

### Bactericidal activity spectrum (time-kill)

At 0.25 µM and 1 h incubation with 16-PLL_10_ log reduction in c.f.u. ml^-1^ of ESKAPE reference strains ranged from 0.62±0.12 for *Enterobacter cloacae* complex NCTC 13405, to at least 5.13±0.13 for *Enterococcus faecium* NCTC 7174 (Table 2). 16-PLL_10_ generally demonstrated higher bactericidal activity against Gram-positive reference strains, compared to Gram-negative strains, except for *K. pneumoniae* NCTC 9633. For *A. baumannii* ATCC 19606, *E. cloacae* complex NCTC 13405, *P. aeruginosa* NCTC 13437*, P. aeruginosa* ATCC 27853, 0.25 µM resulted in less than two log reduction in c.f.u. ml^−1^ but increasing the concentration by ten-fold (2.5 µM), improved activity to >3 log.

**Table 2.** Mean log reduction c.f.u. ml^−1^ among ESKAPE pathogens after 1 h incubation with 16-PLL_10_

		Susceptible reference strain		Resistant strain/isolate	
	Bacterial species	Ref number	Log reductionc.f.u. ml^−1^Mean±SEM*	Ref number	Log reductionc.f.u. ml^−1^Mean±SEM*
Tested at 0.25 µM	*A. baumannii*	19 606	0.65±0.12	CI 23AD06	1.17±0.22
	*P. aeruginosa*	27 853	1.53±0.15	13 437	0.76±0.18
	*E. faecium*	7174	5.13±0.13†	12 204	5.03±0.14
	*K. pneumoniae*	9633	3.05±0.14	2146	5.61±0.05†
	*S. aureus*	25 923	2.42±0.66	43 300	2.23±0.18
	*E. cloacae*	–	–	13 405	0.62±0.14
Tested at 2.5 µM	*A. baumannii*	19 606	4.49±0.39†	CI 23AD06	4.96±0.11†
	*P. aeruginosa*	27 853	4.65±0.10†	13 437	3.70±0.49†
	*E. cloacae*	–	–	13 405	4.60±0.33†

a a*Assays carried out in triplicate on three separate occasions. limit of detection of the assay reached for at least one replicate.

†Limit of detection of the assay reached for at least one replicate.

The bactericidal activity of 16-PLL_10_ was retained among ESKAPE strains and clinical isolates with multidrug resistant phenotypes (Table 2). Most species demonstrated near-equivalent log reductions for resistant and susceptible strains. One exception was *K. pneumoniae* NCTC 9633 (antibiotic-susceptible), with mean log reduction of 3.05±0.14 c.f.u. ml^−1^, compared to 5.61±0.05 for the New Delhi Beta-Lactamase 1 (NDM-1) producing strain, ATCC 2146. Notably, for most bacteria, the assay limit was approached at 2.5 µM, prohibiting inter-isolate comparison of bactericidal activity. For these bacteria, lowering the test concentration to 0.25 µM, although lowering killing overall, allowed discrimination between bactericidal effects. However, under these conditions the effects of AMR phenotype on susceptibility to 16-PLL_10_ were variable.

Nine clinical isolates were selected for investigation, seven from patients with diabetic foot infections. In addition, *A. baumannii* from a bronchoalveolar lavage was a representative multidrug resistant isolate. As streptococci are common wound pathogens, non-ESKAPE isolate, an *S. dysgalactiae* wound isolate was also tested. Bactericidal activity of 16-PLL_10_ against these isolates are summarized in Table 3. Log reductions ranged from 0.30±0.10 for *E. cloacae complex* to 3.81±0.10 for *Acinetobacter lwoffii*. A clinical isolate of vancomycin resistant *E. faecium* (VRE) was susceptible to the lower concentration (0.25 µM) of 16-PLL_10_ (3.01±0.65 log).

**Table 3.** Mean log reductions (c.f.u. ml^−1^) among clinical isolates after 1 h incubation with 16PLL_10_

16PLL_10_**concn**	Species	Ref. no	Site	Underlying condition	Log reductionc.f.u. ml^−1^±SEM*
0.25 µM	*A. baumannii*	23AD06	BAL	–	1.17±0.22
	*A. lwoffii*	22AD05	Foot Ulcer	Diabetes	3.81±0.10
	*E. cloacae*	23AD01	BKA Wound	Diabetes/PVD	0.30±0.10
	*E. faecium* (VRE)	23AD04	Foot Ulcer	Diabetes/PVD	3.01±0.65†
	*K. oxytoca*	23AD11	Foot Ulcer	Diabetes	2.45±0.10
	*P. aeruginosa*	22AD01	Ankle Ulcer	PVD	1.30±0.12
	*S. aureus* (MRSA)	23AD02	Necrotic Toe	Diabetes	2.49±0.17
	*S. aureus* (MSSA)	23AD10	Foot Ulcer	Diabetes	3.22±0.55
	*S. dysgalactiae*	23AD09	Foot Ulcer	Diabetes/Chemotherapy	0.78±0.07
2.5 µM	*A. baumannii*	23AD06	BAL	–	4.96±0.11†
	*E. cloacae*	23AD01	BKA Wound	Diabetes/PVD	4.53±0.36†
	*P. aeruginosa*	22AD01	Ankle Ulcer	PVD	5.53±0.05†
	*S. dysgalactiae*	23AD09	Foot Ulcer	Diabetes/Chemotherapy	3.78±0.29

a *Values shown are the mean±SEM from assays carried out in triplicate on three separate occasions. Reaching limit of assay for some/all experiments at this concentration. BAL – Bronchoalveolar Lavage, BKA – Below Knee Amputation, PVD – Peripheral Vascular Disease

†Reaching limit of assay for some/all experiments at this concentration.

BALbronchoalveolar LavageBKAbelow knee amputationPVDperipheral vascular disease

In clinical isolates with less than two-log reduction at 0.25 µM 16-PLL_10_, increasing the concentration ten-fold, gave greater than 4.5 log reduction in c.f.u. ml^−1^ for the three Gram-negatives. At this concentration, the lowest mean log reduction in c.f.u. ml^−1^ was observed for *S. dysgalactiae* (3.78±0.29).

### Minimum bactericidal concentration (MBC)

MBCs for antibiotic-susceptible and -resistant reference strains are shown in Table 4. *S*. *aureus* ATCC 25923 had the lowest MBC of 2.5 µM, followed by *E. faecium* NCTC 7174 with MBC=5 µM. Gram-negative organisms had higher MBCs ranging from 10 µM for *P. aeruginosa*, to >40 µM for *E. cloacae* complex NCTC 13405. For antibiotic-resistant ESKAPE pathogens, similar MBC disparities between Gram-positive and Gram-negatives were found except for *A. baumannii* strains. *E. cloacae* complex was poorly susceptible with MBC exceeding the highest concentration tested. Comparing MBCs for the antibiotic-resistant and susceptible ESKAPE strains, no consistent pattern was observed in predicting bactericidal activity of 16-PLL_10_. 16-PLL_10_ was bactericidal at a lower concentration for the AMR isolates compared to the susceptible isolates for *A. baumannii*, and *K. pneumoniae*, MBC was equivalent for *S. aureus* strains, independent of resistance phenotype, and was one dilution higher for the ESBL/VIM producing pseudomonas compared to the susceptible isolate.

**Table 4.** Minimum bactericidal concentrations (MBC) and selectivity indices (SI) for 16-PLL_10_ and 25% TFA-16-PLL_10_ against antibiotic-susceptible and resistant bacteria

	Bacteria name	Ref no./isolate	16-PLL_10_	16-PLL_10_	TFA-16-PLL_10_	TFA-16-PLL_10_
			MBC(μM)	SI_IC50_/SI_MHC_^*^	MBC (μM)	SI_IC50_/SI_MHC_*
Susceptible strains	*A. baumannii*	ATCC 19606	20	23.5/32	20	14.3/32
	*E. faecium*	NCTC 7174	5	5.9/8	5	3.6/8
	*K. pneumoniae*	NCTC 9633	25	29.4/40	20	14.3/32
	*P. aeruginosa*	ATCC 27853	10	11.7/16	10	7.1/16
	*S. aureus*	ATCC 25923	2.5	2.9/4	10	7.1/16
Resistant strains	*A. baumannii*	CI 23AD06	1.25	1.47/2	2.5	1.8/4
	*E. faecium*	NCTC 12204	10	11.7/16	10	7.1/16
	*K. pneumoniae*	ATCC 2146	20	23.5/32	>40	>28.5/>64
	*P. aeruginosa*	NCTC 13437	20	23.5/32	40	28.5/64
	*S. aureus*	ATCC 43300	2.5	2.9/4	10	7.1/16
All strains†	–	–	7.8‡	9.1/12.4	13.2‡	9.4/21.1

*Selectivity index calculated by dividing MBC by IC_50_ value (0.85 µM - 16-PLL_10_, 1.4 µM TFA-16-PLL_10_) and MHC_5%_ (0.625 µM).

†Selectivity index calculated by dividing the geometric mean of MBC by IC_50_ value or MHC value.

‡Geometric mean of MBC.

ATCCAmerican tissue culture collectionMBCminimum bactericidal concentrationMHCminimum haemolytic concentration that caused 5% haemolysisNCTCNational Collection of Type CulturesSI_IC50_selectivity index based on IC_50_SI_MHC_selectivity index based on MHC

MBC were performed for all nine clinical isolates and ranged from ≤0.156 µM for *P. aeruginosa* from an infected ankle ulcer in a patient with peripheral vascular disease to >40 µM for *E. cloacae* complex from an infected foot ulcer in a patient with a history of diabetes and peripheral vascular disease. A clinical isolate of *K. oxytoca* from a DFU had MBC of 40 µM and both *Acinetobacter* species tested, had MBCs of 1.25 µM (Table 5). Compared to reference strains, clinical isolates of the same species had similar MBCs to their ESKAPE reference strain counterparts. Only methicillin susceptible *S. aureus* (MSSA) had a MBC greater than one dilution of that of the reference strain. *Pseudomonas* and *Acinetobacter* clinical isolates had MDR phenotypes (Table 2) but MBCs of 16-PLL_10_ were considerably lower than the reference strains.

**Table 5.** Minimum bactericidal concentrations (MBC) and selectivity indices (SI) for 25% TFA-16-PLL_10_ and 16-PLL_10_ for a range of clinical isolates recovered from wounds

Bacteria name	ID no.	Site	16-PLL_10_	16-PLL_10_	TFA-16-PLL^_10_^	TFA-16-PLL_10_
			MBCμM	SI_IC50_/SI_MHC_^*^–	MBCμM	SI_IC50_/SI_MHC_^*^–
*Acinetobacter baumannii*	23AD06	BAL	1.25	1.5/2	2.5	1.8/4
*Acinetobacter lwoffii*	22AD05	Foot Ulcer	1.25	1.5/2	1.25	0.9/2
*E. faecium* (VRE)	23AD04	BKA Wound	5	5.9/8	2.5	1.8/4
*Enterobacter cloacae* complex	23AD01	Foot Ulcer	>40	>47/>64	>40	>28.6/>64
*Klebsiella oxytoca*	23AD11	Foot Ulcer	40	47/64	>40	>28.6/>64
*Pseudomonas aeruginosa*	22AD01	Ankle Ulcer	≤0.156	<0.18/<0.25	≤0.156	<0.11/<0.25
*S. aureus* (MRSA)	23AD02	Necrotic Toe	5	5.9/8	2.5	1.8/4
*S. aureus* (MSSA)	23AD10	Foot Ulcer	10	11.7/16	10	7.2/16
*Streptococcus dysgalactiae*	23AD09	Foot Ulcer	2.5	2.9/4	2.5	1.8/4
All isolates†	–	–	4.3‡	5.0/6.8	4.0‡	2.8/6.3

MBC - , VRE - Vancomycin resistant enterococci, MRSA - Methicillin resistant , MSSA - Methicillin susceptible , MHC – Minimum haemolytic concentration that caused haemolysis, *SI_IC50_ - selectivity index based on IC_50_, SI_MHC_ - selectivity index based on MHC. Selectivity index calculated by dividing MBC by IC_50_ value (0.85 µM - 16-PLL_10_, 1.4 µM TFA-16-PLL_10_) and MHC_5%_ (0.625 µM). Selectivity index calculated by dividing the geometric mean of MBC by IC value or MHC value. Geometric mean of MBC (where MBC and, values of and used in calculation of geometric mean.

†Selectivity index calculated by dividing the geometric mean of MBC by IC_50_ value or MHC value.

‡Geometric mean of MBC (where MBC >40 µM and ≤0.156 µM, values of 80 µM and 0.156 µM used in calculation of geometric mean).

MBCminimum bactericidal concentrationMHCminimum haemolytic concentration that caused 5% haemolysisMRSAmethicillin resistant *S. aureus*MSSAmethicillin susceptible *S. aureus*VREvancomycin resistant enterococci

### Biocompatibility testing of modified 16-PLL_10_; human keratinocyte cytotoxicity and erythrocytes haemolysis

**Fig. 1.**
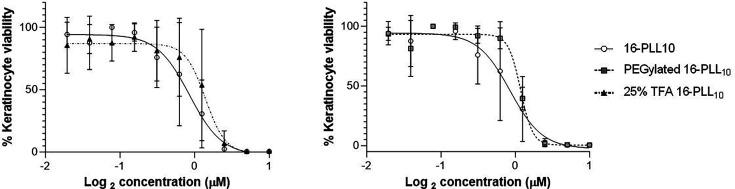
Dose-response curves of HaCaT metabolic activity vs. log concentration of 16-PLL_10_ compared to PEGylated 16-PLL_10_ and TFA 16-PLL_10_. HaCaT cells were seeded to 96 well plates and incubated until a confluent monolayer formed. Cells were treated with 16-PLL_10_, PEGylated 16-PLL_10_ or TFA 16-PLL_10_ (0 to 10 µM) and incubated for 18 h. Cell viability following treatment was determined based on metabolic activity using the MTT assay. Data points shown are the mean±SEM for three experiments carried out in duplicate. IC_50_ values determined from curves were; 0.85 µM (16-PLL_10_), 1.18 µM (PEGylated 16-PLL_10_) and 1.40 µM (TFA 16-PLL_10_).

**Fig. 2.**
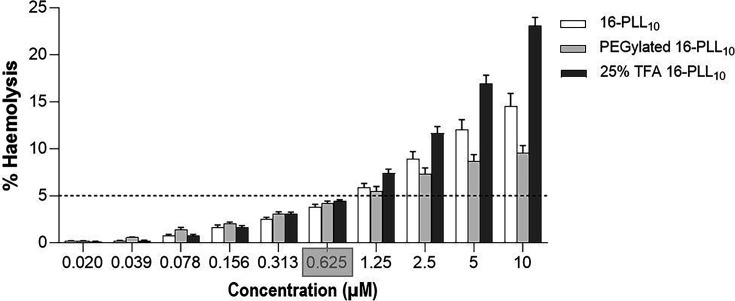
Haemolytic activity of 16-PLL_10_ compared to PEGylated 16-PLL_10_ and TFA 16-PLL_10_. Percentage haemolysis of healthy human erythrocytes exposed to serial doubling concentrations, 0–10 µM of 16-PLL_10_ (white), PEGylated 16-PLL_10_ (light grey), and 25% TFA-16-PLL_10_ (dark grey) for 18 h. Data points shown are the mean±SEM for three experiments carried out in duplicate. Dashed line indicates 5% haemolysis. Grey shading on x axis indicates the MHC_5%_.

16-PLL_10_ was toxic to human cells at antimicrobial concentrations, likely associated with its high positive charge. TFA conjugation of 25% of lysine residues on arms of 16-PLL_10_ aimed to reduce cytotoxicity but retain antimicrobial activity by lowering net positive charge (Table 1). TFA modification resulted in a relatively small but not statistically significant reduction in cytotoxicity to HaCaT cells compared to 16-PLL_10_. IC_50_ value from dose-response curve for TFA-16-PLL_10_ was 1.40 µM, 95% CI (1.06–1.82) compared to 0.85 µM, 95% CI (0.64–1.14) for 16- PLL_10_ (Fig. 1). The second modification aimed at improving biocompatibility with human tissues was PEGylation to mask 25% of the poly-lysine side arms of 16-PLL_10_ (Table 1). Similar to TFA modification, PEGylated 16-PLL_10_ demonstrated improved *in vitro* biocompatibility with human keratinocytes when compared to unmodified 16-PLL_10_. The IC_50_ value was 1.18 µM, 95% CI (1.04–1.30) for PEGylated 16-PLL_10_ compared to 0.85 µM, 95% CI (0.64–1.14) for 16-PLL_10_. Comparing both modifications, the TFA-modified 16-PLL_10_ had a better cytotoxicity profile compared to PEGylated 16-PLL_10_ (IC_50_ value 1.4 µM vs. 1.18 µM) but the difference was not statistically significant. Further characterisation of TFA-16-PLL_10_ and PEGylated 16-PLL_10_ biocompatibility compared to unmodified 16-PLL_10_ was investigated using haemolysis assays. At the highest concentration tested (10 µM), TFA-16-PLL_10_ was the most haemolytic (23.1% haemolysis of erythrocytes compared to 14.5% for 16-PLL_10_ and 9.6% for PEGylated 16-PLL_10_). At concentrations below 1.25 µM, there was no significant difference in haemolysis induced by 16-PLL_10_ vs. modified forms but above this concentration, PEGylated 16-PLL_10_ was better tolerated by erythrocytes than 16-PLL_10_ or TFA-16-PLL_10_. Minimum haemolytic concentration (MHC_5%_), defined as the lowest concentration to cause up to 5% haemolysis was 0.625 µM for 16-PLL_10_, PEGylated 16-PLL_10_ and TFA-16-PLL_10_ (Fig. 2). The ratios of MBC to IC_50_ and MHC_5%_ values were used to calculate selectivity indices (SI), for direct comparison of cell selection for 16-PLL_10_ and TFA-16-PLL_10_. Using the geometric mean of MBCs, SI_MHC_ for reference strains was greater for TFA-16-PLL_10_ compared to 16-PLL_10_ (21.4 vs. 12.1) but SI_IC50_ were similar. However for clinical isolates, SI_MHC_ were similar but TFA-16-PLL_10_ had a lower SI_IC50_ (2.8 vs. 5.0) (Tables4  5).

### Effect of TFA modification of 16-PLL_10_ on bactericidal activity

Given that 25% TFA modified 16-PLL_10_ demonstrated moderately less cytotoxicity to keratinocytes than 16-PLL_10_ and was only marginally more haemolytic, the effect of this modification on MBCs was investigated for reference strains and clinical isolates (Table 5).

For reference strains, the MBC for 25% TFA-16-PLL_10_ was the same or one dilution higher/lower compared to the MBC for unmodified 16-PLL_10_, except for *S. aureus*, ATCC 25923 (MSSA), ATCC 43300 (MRSA), and *K. pneumoniae* ATCC 2146. For these, modified 16-PLL_10_ had four-fold higher MBC than unmodified (10 µM vs. 2.5 µM). This was not observed for clinical strains (Table 5). Bactericidal time-kill assays were performed for selected reference strains. For *S. aureus* ATCC25923 incubation with unmodified 16-PLL_10_, 0.25 µM, 1 h, resulted in mean log reduction of 2.56±0.16 compared to 25% TFA-16-PLL_10_ (0.13±0.07). This was statistically significant, *P*<0.001 (Fig. S1). For *E. faecium* NCTC 7174, activity remained high for TFA-16-PLL_10_ compared to 16-PLL_10_ (mean log reductions of 5.21±0.08 and 5.13±0.13, respectively at 0.25 µM). For * K. pneumoniae* ATCC 2146 (carbapenemase-producing enterobacterales (CPE) NDM-1), 25% TFA-16-PLL_10_ also demonstrated reduced killing compared to 16-PLL_10_, which was statistically significant, with a mean log reduction of 3.87±0.44, compared to 5.61±0.05, *P*<0.01. At 0.25 µM, killing of *P. aeruginosa* ATCC 27853 was too low to establish differences between modified and unmodified 16-PLL_10_. However, potent killing beyond the assay limits, at 2.5 µM precluded the discernment of differential activity.

## Discussion

In our study, 16-PLL_10_ had broad bactericidal activity against ESKAPE strains and isolates from patients with wound infections. However, we cannot recommend progression of these specific star AMPs towards clinical trials because even with modification, they are unfavourably toxic to human keratinocytes, and erythrocytes. While substantial efforts to progress AMPs to clinical use have occurred (e.g. Pexiganan), where antibiotic options are limited there has been little success in making topical AMPs available for patients [22,23]. Publications to date re-iterate claims of AMP broad-spectrum activity, though laboratory studies rarely demonstrate this for more than a few reference strains. Less is published demonstrating the activity of PLLs against clinical isolates, and relatively few investigators have considered the activity of synthetically engineered polymeric PLLs. However, more recent publications, have purposefully demonstrated bactericidal activity against clinically relevant pathogens, usually limited to one representative Gram-positive and Gram-negative pathogen, typically strains of *E. coli*, *S. aureus* and *P. aeruginosa* [13,14, 16]. To our knowledge, no studies have demonstrated the spectrum of polymeric PLLs against a broad range of susceptible and resistant reference strains and clinical isolates. By selecting a broad range of clinically-important bacteria, including AMR strains, we demonstrated that star PLL-based AMPs generally had lower MIC and MBC for Gram-positive compared to Gram-negative reference strains, though with some exceptions (*Acinetobacter* clinical isolates). These MBCs approximated closely with MICs reported by Lu *et al*. for their PLL based star AMPs [13]. For a structurally similar, 15-arm PLL_10_, Lu *et al.* described MICs for MRSA, methicillin resistant *Staphylococcus epidermidis*, *P. aeruginosa*, and *A. baumannii* reference strains of 2.2 µM, 1.1 µM, 8.8 µM, and 8.8 µM, respectively. Our 16-PLL_10_ time-kill assays, achieved broad spectrum killing of ESKAPE pathogens at 2.5 µM independent of susceptibility phenotype. Notably, 16-PLL_10_ had potent activity against extensively resistant strains and clinical isolates such as a CPE NDM-1 producing *K. pneumoniae* (susceptible to colistin only), and MDR *A. baumannii* clinical isolate.

Establishing the therapeutic index between the concentrations at which bactericidal activity and human cytotoxicity occurs, is essential in the development of new bactericidal antibiotics. It appears that for 16-PLL_10_ the therapeutic index is likely to be narrow. Based on the comparative cytotoxicity of various PLLs with different arm lengths and number of arms, Walsh *et al.* speculated, that lower cytotoxicity of a 64 arm PLL with shorter arm length of five lysine residues (64-PLL_5_), correlated with lower density of positive charge encountered by eukaryotic cell membranes afforded by shorter arms (rather than number of arms) [17]. Meanwhile Lu *et al.* determined that increases in PLL arm length and density of PLL based star AMPs could increase the surface electrostatic potential, which increased affinity for anionic membranes [13]. For the antimicrobial investigation of star-PLLs here, a relatively short arm length of ten lysine residues was selected at the outset, recognizing that positive charge was central to bacterial cell membrane interaction and bactericidal activity. However, cytotoxicity investigations, in relevant cell types, showed that 16-PLL_10_ lacked selective toxicity for bacterial cells. Modifications of 16-PLL_10_ included conjugation with TFA groups, or PEGylation. Conjugation with TFA reduces the net cationic charge of the AMP. This was previously employed to facilitate better intracellular penetration for mammalian cell gene delivery [24]. PEGylation is utilised to enhance biocompatibility of drugs by protecting or shielding drugs from unwanted interactions with human biomolecules. PEGylation in tandem with acetylation/fluorination has been utilised for gene delivery, and had lower cytotoxicity and increased AMP stability [25]. Here, 16-PLL_10_ modified by PEGylation was employed to improve biocompatibility by masking the active lysine side arms from interactions with the human cell membrane [26,27]. The modifications to 16-PLL_10_ only moderately improved its human cell cytotoxicity profile. Considering the MBCs for all but one of the 19 bacterial isolates or reference strains tested, exceeded the IC_50_ and 5% haemolysis, it appears that this AMP, even with modifications is not a useful candidate for progression to clinical use.

The AMPs which most closely resemble those evaluated here, are reported to have good selectivity indices, and the authors drew positive conclusions about the potential of PLL based star AMPs [13,14]. Interestingly, the selectivity index used was based on bactericidal results which were similar to ours, however, the only biocompatibility data included in SI calculation, was based on haemolytic activity in non-human cells. Lu *et al.* demonstrated a half-maximal haemolytic concentration (HC_50_) of >2000 µg ml^−1^ for a similar star-PLL (15-PLL_10_) to murine erythrocytes [13]. Lam *et al.* demonstrated HC_50_ of 58.3 µM for a 16-arm heteropolymeric star-AMP, and 45.3 µM for the 32-arm heteropolymeric star-AMP against ovine erythrocytes [14]. Haemolysis data from these previous publications are not easily compared to the present work due to vastly different approaches used to determine haemolytic concentrations, and the use of non-human mammalian erythrocytes. Others have recognised that non-standardised approaches to determining haemotoxicity, poses a challenge in the progression of AMPs towards clinical applications [28]. To our knowledge, our research is the first to highlight cytotoxicity to human keratinocytes. The only prior preclinical cytotoxicity investigation of AMPs to human cell lines, was by Lam *et al.* using renal cells (HEK293T) and hepatocytes (H4IIE) [14]. While these are informative studies, skin cells lines may better represent the principally envisioned topical clinical application of AMPs.

Among star-AMPs, other approaches to improve biocompatibility include shortening PLL arm length or density, a switch from homopolymers to heteropolymers, or liposomal formulation, as is utilised for some toxic antimicrobials such as amphotericin and colistin [13,14]. There may be further scope to develop less cytotoxic AMPs. While we aimed to achieve this by PEGylation or fluorination, this could be addressed more systematically by utilising computational biology, including machine learning and artificial intelligence (AI), to screen and predetermine the SIs for candidate AMPs [29]. Supporting this, Haney *et al.*, utilised computational modelling, to optimise screening for anti-biofilm peptides. This model was 85% successful, and identified one peptide with an eight-fold increase in anti-biofilm activity over their original peptide [30]. Use in a mouse model of MRSA abscess, showed significant reduction in abscess size. Exploitation of AI to identify AMPs will also require consideration of intracellular mechanisms and targets which may warrant more fundamental research. In addition, further research is required into cytotoxicity, stability, wound healing, anti-inflammatory, and haemostatic effects of AMPs [10,31,34].

Limitations of our study include the absence of a comparator in the bactericidal assays and microbroth dilution experiments. The wide range of bacteria investigated here, would make rationalisation to one or two currently utilised antibiotics difficult. However, we previously showed more potent activity of other PLLs compared to rifampicin and gentamicin [16]. Furthermore, antibiofilm activity was not evaluated against ESKAPE pathogens. However, we previously demonstrated that this PLL series, albeit at higher concentrations (50 µM), has potent antibiofilm activity against *S. aureus* and *P. aeruginosa* isolates recovered from wound infections [16].

In conclusion, although 16-PLL_10_ has broad bactericidal activity, it is unfavourably toxic to human keratinocytes, and erythrocytes. Attempts to modify 16-PLL_10_ did not substantially affect bactericidal activity, but neither did it significantly reduce cytotoxicity to human cells. We cannot recommend the further clinical progression of these star AMPs in their current form. However, on consideration of patient need and the clinical gaps that continue to persist, it is notable that 16-PLL_10_ was bactericidal against AMR reference strains and isolates for which there are currently almost no satisfactory antimicrobial options available. Given the scale of the AMR crisis for global healthcare and society, we believe that despite the challenges, further work on progressing AMPs including structurally engineered nanomaterials, for clinical use is necessary. Characterisation studies reporting both positive and negative biological properties can better inform the development of robust data training sets to support machine learning for drug discovery. Furthermore, financial and organisational support required to ensure the advancement of more standardised and collaborative approaches to performing and disseminating research on AMPs should be advocated for.

## supplementary material

10.1099/jmm.0.001886 Uncited Supplementary Material 1.

## Abbreviations

AMP: antimicrobial peptide
AMR: antimicrobial resistance
ATCC: American Type Culture Collection
BSA: bovine serum albumin
CPE: carbapenemase producing enterobacterales
DFU: diabetic foot ulcer
DMEM: Dulbecco’s modified Eagles medium
DMSO: dimethyl sulfoxide
ESKAPE: *Enterococcus* spp., *Staphylococcus aureus*, *Klebsiella pneumoniae, Acinetobacter baumannii, Pseudomonasaeruginosa, Enterobacter* spp.)
FBS: foetal bovine serum
MALDI-TOF MS: Matrix Associated Laser Ionisation Desorption Time-of-FlightMass Spectrometry
MBC: minimum bactericidal concentration
MH: Mueller Hinton
MHC: minimum haemolytic concentration
MRSA: methicillin resistant *S. aureus*
MSSA: methicillin susceptible *S. aureus*
MTT: 3-(4,5-dimethylthiazol-2-yl)−2-,5-diphenyltetrazoliumbromide
NCTC: National Collection of Type Cultures
PBS: phosphate buffered saline
PEG: Poly-ethyleneglycol
PLL: poly-l-lysine
SI: selectivity index
SNAPP: structurally nanoengineered antimicrobial peptide polymer
TFA: trifluoroacetylated
UKHSA: United Kingdom Health Security Agency
VRE: vancomycin resistant *E. faecium*

## References

[1] Antimicrobial Resistance Collaborators (2022). Global burden of bacterial antimicrobial resistance in 2019: a systematic analysis. Lancet.

[2] Bader MS (2008). Diabetic foot infection. Am Fam Physician.

[3] Senneville É, Albalawi Z, van SA, Abbas ZG, Allison G (2023). IWGDF/IDSA guidelines on the diagnosis and treatment of diabetes-related foot infections (IWGDF/IDSA 2023). Clin Infect Dis.

[4] Surgical site infections: prevention and treatment. https://www.ncbi.nlm.nih.gov/books/NBK542473.

[5] (2018). WHO Prevention and Management of Wound Infection Guideline, second edition. https://www.who.int/publications/i/item/prevention-and-management-of-wound-infection.

[6] Shen W, He P, Xiao C, Chen X (2018). From antimicrobial peptides to antimicrobial poly(α-amino acid)s. Adv Healthc Mater.

[7] Shai Y (1999). Mechanism of the binding, insertion and destabilization of phospholipid bilayer membranes by alpha-helical antimicrobial and cell non-selective membrane-lytic peptides. Biochim Biophys Acta.

[8] Matsuzaki K (1999). Why and how are peptide-lipid interactions utilized for self-defense? Magainins and tachyplesins as archetypes. Biochim Biophys Acta.

[9] Yang L, Weiss TM, Lehrer RI, Huang HW (2000). Crystallization of antimicrobial pores in membranes: magainin and protegrin. Biophys J.

[10] Patil NA, Kandasubramanian B (2021). Functionalized polylysine biomaterials for advanced medical applications: a review. Eur Polym J.

[11] Ramamurthy R, Mehta CH, Nayak UY (2021). Structurally nanoengineered antimicrobial peptide polymers: design, synthesis and biomedical applications. World J Microbiol Biotechnol.

[12] Zheng M, Pan M, Zhang W, Lin H, Wu S (2021). Poly(α-l-lysine)-based nanomaterials for versatile biomedical applications: current advances and perspectives. Bioact Mater.

[13] Lu C, Quan G, Su M, Nimmagadda A, Chen W (2019). Molecular architecture and charging effects enhance the *in vitro* and *in vivo* performance of multi‐arm antimicrobial agents based on star‐shaped poly(l‐lysine). Adv Ther.

[14] Lam SJ, O’Brien-Simpson NM, Pantarat N, Sulistio A, Wong EHH (2016). Combating multidrug-resistant Gram-negative bacteria with structurally nanoengineered antimicrobial peptide polymers. Nat Microbiol.

[15] Walsh DP, Murphy RD, Panarella A, Raftery RM, Cavanagh B (2018). Bioinspired star-shaped poly(l-lysine) polypeptides: efficient polymeric nanocarriers for the delivery of DNA to mesenchymal stem cells. Mol Pharm.

[16] Grace A, Murphy R, Dillon A, Smith D, Cryan S-A (2022). Modified poly(L-lysine)-based structures as novel antimicrobials for diabetic foot infections, an *in-vitro* study. *HRB Open Res*.

[17] Walsh DP, Raftery RM, Murphy R, Chen G, Heise A (2021). Gene activated scaffolds incorporating star-shaped polypeptide-pDNA nanomedicines accelerate bone tissue regeneration *in vivo*. Biomater Sci.

[18] Byrne M, Victory D, Hibbitts A, Lanigan M, Heise A (2013). Molecular weight and architectural dependence of well-defined star-shaped poly(lysine) as a gene delivery vector. Biomater Sci.

[19] Miles AA, Misra SS, Irwin JO (1938). The estimation of the bactericidal power of the blood. J Hyg.

[20] Zapotoczna M, Forde É, Hogan S, Humphreys H, O’Gara JP (2017). Eradication of *Staphylococcus aureus* biofilm infections using synthetic antimicrobial peptides. J Infect Dis.

[21] Oren Z, Shai Y (1998). Mode of action of linear amphipathic alpha-helical antimicrobial peptides. Biopolymers.

[22] Lipsky BA, Holroyd KJ, Zasloff M (2008). Topical versus systemic antimicrobial therapy for treating mildly infected diabetic foot ulcers: a randomized, controlled, double-blinded, multicenter trial of pexiganan cream. Clin Infect Dis.

[23] Silverman MH Pexiganan versus placebo control for the treatment of mild infections of diabetic foot ulcers. A randomized, double-blind, multicenter, superiority, placebo-controlled phase 3 study of pexiganan cream 0.8% applied twice daily for 14 days in the treatment of adults with mild infections of diabetic foot ulcers. https://clinicaltrials.gov/ct2/show/record/NCT01590758.

[24] Ge C, Yang J, Duan S, Liu Y, Meng F (2020). Fluorinated α-helical polypeptides synchronize mucus permeation and cell penetration toward highly efficient pulmonary siRNA delivery against acute lung injury. Nano Lett.

[25] Wu T, Wang L, Ding S, You Y (2017). Fluorinated PEG‐polypeptide polyplex micelles have good serum‐resistance and low cytotoxicity for gene delivery. Macromol Biosci.

[26] Morris CJ, Beck K, Fox MA, Ulaeto D, Clark GC (2012). Pegylation of antimicrobial peptides maintains the active peptide conformation, model membrane interactions, and antimicrobial activity while improving lung tissue biocompatibility following airway delivery. Antimicrob Agents Chemother.

[27] Veronese FM, Harris JM (2002). Introduction and overview of peptide and protein pegylation. Adv Drug Deliv Rev.

[28] Greco I, Molchanova N, Holmedal E, Jenssen H, Hummel BD (2020). Correlation between hemolytic activity, cytotoxicity and systemic in vivo toxicity of synthetic antimicrobial peptides. Sci Rep.

[29] Ruiz Puentes P, Henao MC, Cifuentes J, Muñoz-Camargo C, Reyes LH (2022). Rational discovery of antimicrobial peptides by means of artificial intelligence. Membranes.

[30] Haney EF, Straus SK, Hancock REW (2019). Reassessing the host defense peptide landscape. Front Chem.

[31] Zarrintaj P, Ghorbani S, Barani M, Singh Chauhan NP, Khodadadi Yazdi M (2022). Polylysine for skin regeneration: a review of recent advances and future perspectives. Bioeng Transl Med.

[32] Haney EF, Brito-Sánchez Y, Trimble MJ, Mansour SC, Cherkasov A (2018). Computer-aided discovery of peptides that specifically attack bacterial biofilms. Sci Rep.

[33] Mookherjee N, Anderson MA, Haagsman HP, Davidson DJ (2020). Antimicrobial host defence peptides: functions and clinical potential. Nat Rev Drug Discov.

[34] de la Fuente-Núñez C, Hancock REW (2015). Using anti-biofilm peptides to treat antibiotic-resistant bacterial infections. Postdoc J.

