# Supplementary material for: Antimicrobial spectrum against wound pathogens and cytotoxicity of star-arranged poly-l-lysine-based antimicrobial peptide polymers
Source: J Med Microbiol. 2024 Sep 13;73(9):001886. doi: 10.1099/jmm.0.001886 (PMC11394093; doi:10.1099/jmm.0.001886)
Supplement: Uncited Supplementary Material 1. [file jmm-73-01886-s001.pdf]

Antimicrobial activity spectrum against wound pathogens and cytotoxicity evaluation of poly-L-lysine-based antimicrobial peptide polymers in star-arrangements

Aaron Doherty [1,3,4], Robert Denis Murphy [2], Andreas Heise [2], Fidelma Fitzpatrick [1,3], Deirdre Fitzgerald-Hughes [1*]

[1]Department of Clinical Microbiology, Royal College of Surgeons in Ireland University of Medicine and Health Sciences, Education and Research Centre, Beaumont Hospital, Dublin 9, Ireland.

[2]Department of Chemistry, Royal College of Surgeons in Ireland University of Medicine and Health Science, 123 St. Stephen's Green, Dublin 2, Ireland.

[3]Department of Clinical Microbiology, Beaumont Hospital, Dublin 9, Ireland.

[4]Department of Clinical Microbiology, Cork University Hospital, Wilton, Cork, Ireland

***Corresponding author and email address**

dfitzgeraldhughes@rcsi.ie: Department of Clinical Microbiology, Royal College of Surgeons in Ireland, Dublin, Ireland, Telephone number: 353 1 8093711

Supplemental Table S1 Antimicrobial susceptibilities and resistances for bacterial reference strains and clinical isolates

	Species	Reference ID	Resistances	Susceptibilities
Reference strains	*A. baumannii*	ATCC19606	N/A	AMK, CAZ, CIP, CN, MEM, TOB
	E. cloacae complex (*AmpC* only)	NCTC 13405	AMC, AMP, FOX	AMK, CAZ, CIP, CN, COL, CTX, ERT, IMP, MEM, TIG, TOB, TZP,
	E. faecium (VRE)	NCTC 12204	AMP, TEC, VAN	DAP, LNZ, Q-D
	E. faecium	NCTC 7174	N/A	N/A
	K. pneumoniae	NCTC 9633	N/A	N/A
	K. pneumoniae (CPE NDM-1, ESBL)	ATCC 2146	AMK, AMP, AMC, AZM, CAZ, CIP, CN, CPD, CRO, CTX, CXM ERT, FOX, IMP, MEM, NF, TET TIG, TOB, SXT	COL
	P. aeruginosa	ATCC 27853	N/A	N/A
	P. aeruginosa (VIM/ESBL)	NCTC 13437	AMK, AZM, CAZ, CIP, CN, IMP MEM, TOB, TZP	COL
	S. aureus (MRSA)	ATCC 43300	MET, OX	
	S. aureus	ATCC 25923	N/A	N/A
	Species	**Site of infection/ Underlying disease**		
Clinical Isolates	*P. aeruginosa*	Ankle ulcer right/PVD	Not Tested	Not Tested
	A. lwoffi	Right foot ulcer/Diabetes	CIP	AMK, CN, MEM, TOB
	E. cloacae complex	Ulcer foot/Diabetes/PVD	AMK, AZM, CAZ, CIP, CN, CRO, ERT, MEM, TZP	AMP, AMC
	S. aureus (MRSA)	Pus Toe/Diabetes	CIP, CN, DAP, LNZ, MUP, SXT, TEC, VAN	CLI, ERY, FOX, OX, TET
	E. faecium (VRE)	BKA/Diabetes/PVD	AMP, VAN	LNZ

A. baumannii	BAL/unknown	AZM, CIP, CN, MEM, TIG, TZP, SXT	AMK, FDC
S. dysgalactiae	Foot Ulcer/Diabetes/Oncology	-	ERY, PEN, TET, SXT, VAN
S. aureus	Foot Ulcer/Diabetes	-	CIP, CLI, CN, ERY, FOX, MUP, LNZ, OX, TET, SXT, VAN
K. oxytoca	Foot Ulcer/Diabetes	AMP	AMC, AMK, AZM, CAZ, CIP, CN, CRO, CXM, ERT, FOS, MEM, SXT, TZP

AMC – Amoxicillin-Clavulanate, AMP – Ampicillin, AMK – Amikacin, AZM – Aztreonam, CAZ – Ceftazidime, CIP – Ciprofloxacin, CN – Gentamicin, COL – Colistin, CRO – Ceftriaxone, CTX – Cefotaxime, CXM – Cefuroxime, DAP – Daptomycin, ERT – Ertapenem, FOX – Cefoxitin, LNZ – Linezolid, MEM – Meropenem, OX – Oxacillin(/Flucloxacillin), Q-D – Quinpristin-Dalfopristin, SXT – Trimethoprim-sulfamethoxazole, TEC – Teicoplanin, TET – Tetracyclines, TIG – Tigecycline, TOB – Tobramycin, TZP – Piperacillin-Tazobactam, VAN – Vancomycin, ERY – Erythromycin (Macrolides) FDC – Cefiderocol, FOS – Fosfomycin, MUP- Mupirocin, BAL – Bronchoalveolar Lavage, BKA – Below Knee Amputation, PVD - Peripheral Vascular Disease, ATCC – American Type Culture Collections, NCTC – National Collection of Type Cultures

Supplemental Figure S1

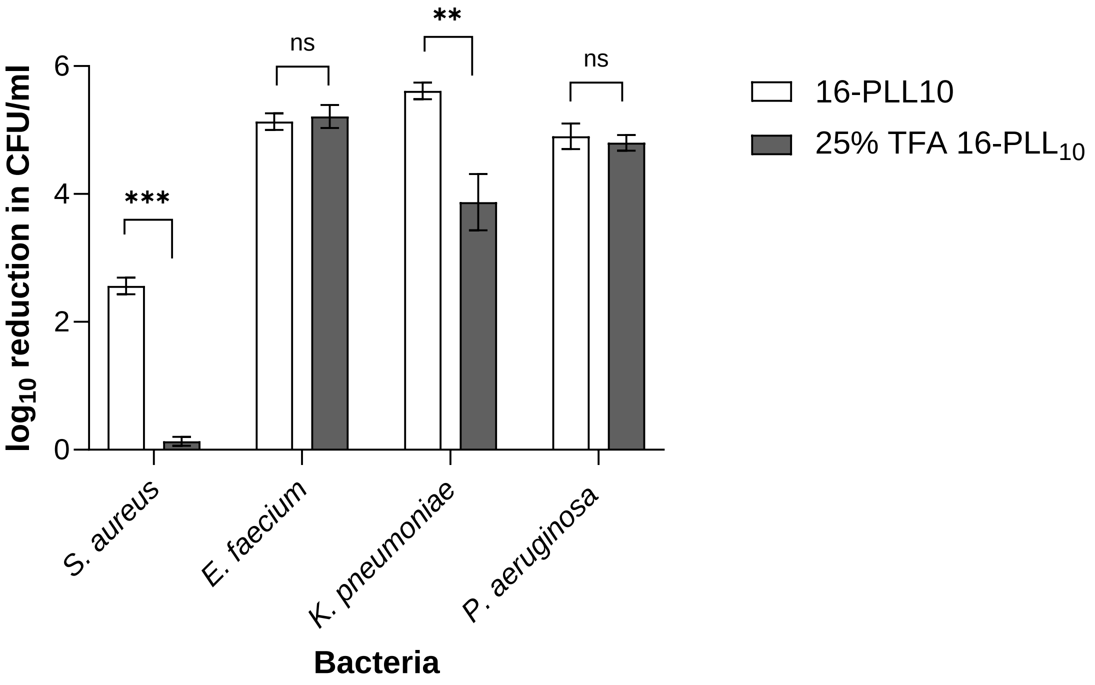

Figure S1 Comparison of bactericidal activity of 16-PLL$_{10}$ and 25% TFA conjugated 16-PLL$_{10}$ against two gram-positive and two gram-negative reference strains.

Bacteria at an approximate concentration of 5 log$_{10}$ CFU/mL were incubated with 0.25µM 16-PLL$_{10}$ or 25% TFA-16-PLL$_{10}$ (*S. aureus, E. faecium, K. pneumoniae*) or 2.5µM (*P. aeruginosa*) in 10 mM Potassium Phosphate Buffer containing 0.2 % BSA for 1h. Data shown are the log reduction in CFU/ml compared to those incubated in the absence of 16-PLL$_{10}$. Values are the mean ± SEM for assays carried out in duplicate on at least three separate occasions. Students t-test was used to determine statistical significance, NS = $p>0.05$, **= $p \leq 0.01$., *** = $p \leq 0.001$
